# Fetal size, gestational age, and cognitive performance at 5 years in term‐born children: Four national cohorts' study

**DOI:** 10.1002/ijgo.70671

**Published:** 2025-11-17

**Authors:** Xuan Zhao, Robert Eves, Siobhan Quenby, Sakari Lemola, Dieter Wolke

**Affiliations:** ^1^ Department of Psychology, Lifespan Health and Wellbeing Group University of Warwick Warwick UK; ^2^ Warwick Medical School University of Warwick Warwick UK; ^3^ Fakultät für Psychologie und Sportwissenschaft Universität Bielefeld Bielefeld Germany

**Keywords:** appropriate for gestational age, cognitive outcomes, early‐term, fetal size, gestational age, intelligence quotient, large for gestational age, small for gestational age, timing of delivery

## Abstract

**Objective:**

Fetal size and gestational age are essential factors to consider when determining the timing of delivery between mothers and obstetricians in term pregnancies. Previous studies have shown that both fetal size and gestational age have associations with cognitive or academic outcomes. This study aimed to determine whether the association between gestational age (37–41 weeks) and child intelligence is moderated by fetal size in term‐born children.

**Methods:**

Data were harmonized for four national cohorts in the USA, UK, Ireland, and Australia. Predictors included fetal size and gestational age. Fetal size was calculated using Fenton's chart and grouped into three categories: Large for gestational age (LGA) (>90th percentile), appropriate for gestational age (AGA) (10th to 90th percentile), and small for gestational age (SGA) (<10th percentile). The outcome was intelligence quotient (IQ) scores at age 5 years. Linear models, contrast analyses, and point plots were employed.

**Results:**

In total, 30 035 term‐born participants were included in the analysis. Overall, being born before 41 weeks and being born with SGA (but not LGA) were both negatively associated with IQ. No statistically significant interactions between fetal size category and gestational age at term were found. At each gestation from 37 to 41 weeks, being born SGA (but not LGA) was associated with lower IQ when compared to AGA. A small but clinically significant reduction in IQ (i.e., 0.23 IQ *Z*‐score, equivalent to a 3.45 IQ difference) was found in SGA‐born children who were born at 37 weeks compared to 41 weeks.

**Conclusion:**

The association between gestation and child IQ at age 5 was not moderated by fetal size in term‐born children. Regardless of gestational age at term, SGA (but not LGA) is consistently and unfavorably associated with poorer cognitive outcomes. The IQ at age 5 was only clinically meaningfully decreased in SGA children born at 37 weeks.

## 1 INTRODUCTION

Mothers and obstetricians are often faced with the challenging task of deciding on the timing of delivery at term (37^+0^–41^+6^ weeks) for a multitude of reasons relating to the risk of fetal and maternal morbidity such as gestational diabetes, gestational hypertension, intrauterine growth restriction, decreased fetal movements, and risk of complicated emergency cesarean section. A significant proportion of term deliveries, around one in three to four, are the result of elective induction or cesarean section.^1^, ^2^, ^3^ While most clinical obstetric research focuses on the association between the timing of delivery and perinatal outcomes,^4^ it is crucial to remember that long‐term cognitive outcomes are equally important in this decision making process.^5^ The need for comprehensive evidence to guide mothers and obstetricians in making informed choices regarding delivery plans is urgent and cannot be overstated.

It is known that in term‐born children, gestational age^6^, ^7^, ^8^, ^9^, ^10^, ^11^ or fetal size (i.e., weight for gestational age)^6^, ^12^, ^13^ are associated with cognitive outcomes. Children born at 37 weeks had cognitive scores 1.95 points lower than those born at 40 weeks.^14^ Previous registry data analyses have indicated that birth at 41 weeks is associated with the lowest risk of adverse academic outcomes compared to all other gestation age.^6^ Children with large for gestational age (LGA) had cognitive scores 0.90 points higher^14^ but small for gestational age (SGA) 5.10 lower^15^ than those with appropriate for gestational age (AGA). The increase in gestational age and body size reflect the maturity and nutritional status of the fetus, respectively. Gestational age shortening is known to be associated with relative ventricular enlargement and decreased relative gray matter, cerebellum, and brainstem volumes, accompanied by reduced anisotropy fraction and increased mean axial and radial diffusion coefficients in major white matter tracts.^16^ Malnutrition‐induced damage originates from impaired neurogenesis and synaptogenesis, ultimately affecting critical brain regions, including the cerebral cortex and cerebellum.^17^ From a biological perspective, they jointly affect brain growth and development, and therefore may interact with each other. However, the known evidence regarding their conjoint and interactive effects is contradictory,^18^, ^19^, ^20^, ^21^, ^22^, ^23^ particularly lacking in evidence focusing on children born at term. It is unknown how cognitive outcomes are associated with gestational age in term children according to fetal size category; that is, in SGA, AGA, and LGA newborns, respectively. Obstetricians require robust evidence on long‐term outcomes to inform decision making when formulating delivery strategies for different fetal sizes. For example, should early delivery, when the baby's absolute weight is relatively low, be expedited for suspected LGA babies to mitigate the risk of shoulder dystocia?^24^

In the present study we undertook a comprehensive examination of the conjoint associations of fetal size and gestational age on intelligence quotient (IQ) in term‐born children. Utilizing harmonized national cohort data from four countries, the study addressed the following research questions: First, is the association between gestation and IQ moderated by fetal size; that is, are the adverse associations of lower gestation disproportionally larger in one of the fetal size categories? Second, what is the conjoint associations of fetal size and gestational age at term (37–41) on child IQ?

## 2 MATERIALS AND METHODS

### 2.1 Sample and procedure

**FIGURE 1:**
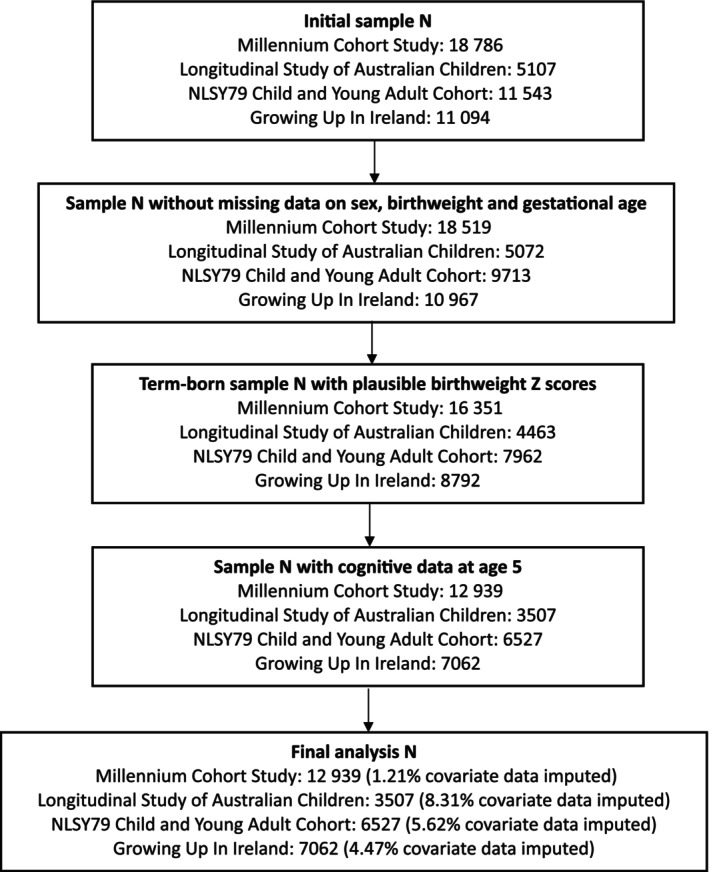
Cohort flow chart.

This study utilized secondary data from four large, longitudinal cohorts in four high‐income, English‐speaking countries. They include the Millennium Cohort Study (MCS)^25^ from the UK, the Longitudinal Study of Australian Children (LSAC)^26^ from the Commonwealth of Australia, the National Longitudinal Survey of Youth 1979 (NLSY79)^27^ from the USA, and the Growing up in Ireland study (GUI)^28^ from the Republic of Ireland. In LSAC and GUI, only participants with available perinatal data were included in this analysis. Written informed consent was obtained from parents, and all studies were reviewed by the institutional review boards or ethics committees.^29^, ^30^, ^31^, ^32^ For sample flow charts and analysis samples, see Figure 1. Additionally, only participants in all cohorts with gestational age at birth between 37^+0^ and 41^+6^ weeks were included in the analysis (see Figure 1).

### 2.2 Gestational age and fetal size

In this study, gestational age refers to the number of completed weeks of pregnancy. Fetal size is represented by the size of the neonate as antenatal estimates were not available across four cohorts (i.e., birth weight *Z*‐score calculated using the Fenton size for gestational age at birth calculator^33^ based on the birth weight, gestational age, and sex of the neonate). All participants' birth weight *Z*‐scores were categorized into the fetal size categories. LGA is defined as fetal size greater than the 90th percentile (i.e., birth weight *Z*‐score > 1.28), while AGA falls between the 10th and 90th percentiles (i.e., birth weight *Z*‐score ≥ −1.28 and ≤1.28), while SGA is below the 10th percentile (i.e., birth weight *Z*‐score < −1.28).

In all four cohorts, information regarding gestational age and birth weight was provided by the primary caregiver. After being verified by matching with official birth records, the parent report data in MCS have been found to be highly accurate.^34^, ^35^ In LSAC, the data were parent‐reported, derived from their equivalent of the parent‐held health record, which was highly likely to have been reviewed by health workers and can be regarded as medical reports. The parent reported data from both other cohorts have not been validated. The participants with birth weights lower than <2500 g in GUI were removed due to the original data being binned, which could introduce inaccuracies in the analysis. Fenton's chart is an international reference developed based on 3 986 456 births from six countries (Germany, the US, Italy, Australia, Scotland, and Canada).^33^ To minimize the impact of reporting errors, participants from all cohorts with a birth weight percentile lower than 0.003% (birth weight *Z*‐score < −4) or greater than 0.998% (birth weight *Z*‐score > 4) were excluded from the analyses.

### 2.3 Cognitive performance ‐ full scale IQ

Fluid (e.g., visual spatial integration) and crystallized (e.g., vocabulary) intelligence were assessed by several standard cognitive tests. The primary outcome is the full‐scale IQ score that was calculated as the average of subtests. The full‐scale IQ score was standardized to a *Z*‐score within each cohort. All measures of cognitive scores using standardized tests were taken at approximately 5 years old, except for fluid intelligence that was measured at 7 years in the LSAC.

Fluid intelligence is the capacity to think speedily and reason flexibly to solve new problems without relying on experience and accumulated knowledge. To measure fluid intelligence in the MCS, the British Ability Scales (BAS) Picture Similarities and Pattern Construction tests^36^ were employed. In the LSAC, the Wechsler Intelligence Scale for Children sixth edition (WISC‐IV)^37^ was used. In the NLSY79, the Peabody individual achievement test (PIAT)^38^ mathematics was administered. In the GUI, the BAS picture similarities test^36^ was taken. Crystallized intelligence is linked to education, experience, and cultural background and is measured by tests of general knowledge such as vocabulary. To assess crystallized intelligence, BAS's naming vocabulary test^36^ was employed in the MCS and GUI. The Peabody picture vocabulary test (PPVT) third edition^39^ was used in the LSAC, while the PPVT revised edition^40^ was used in the NLSY79.

### 2.4 Covariates

Based on previous research that investigated the effects of gestational age or birth weight on IQ,^19^ several variables were selected as covariates in the analysis. They included child sex, maternal age, maternal height, maternal weight, marital status, child's primary language, maternal education, household income (classified as low [bottom 33%], middle [middle 33%], and high [top 33%]), and cohort. Whether English was the child's first language was measured at 5 years. All other covariates were assessed at the nearest assessment point to birth.

### 2.5 Statistical analysis

R version 4.1.1. (R foundation, https://cran.r‐project.org/bin/windows/base/old/4.1.1/) was used to perform all analyses. IQ *Z*‐scores and covariates in the four longitudinal cohorts were harmonized to be comparable. Missing data of covariates only were considered as missing at random and were imputed for the pooled individual participant data analysis of gestation and birth weight on 5‐year IQ. The multiple imputation by chained equations (MICE) algorithm was used to perform the multiple imputation. Gestational age and fetal size were treated as continuous (i.e., for gestational age, gestational weeks, and for fetal size, birth weight *Z*‐score) or categorical variables (i.e., for gestational age, 37, 38, 39, 40, 41 weeks, and for fetal size, LGA, AGA, SGA). To test whether the association between gestation and IQ was moderated by the fetal size, multivariable regressions using generalized least squares were conducted. We ran two models: an unadjusted model with only gestational age and fetal size category, along with their interaction terms, and an adjusted regression model that also included the aforementioned confounders as factors associated with child IQ at year 5. In addition, to test for the conjoint association of gestation with IQ within each fetal size category and to explore what combination of gestational age at term and fetal size category would be associated with IQ reduction by a clinically meaningful magnitude (i.e., difference of IQ *Z*‐score > 0.2 equivalent to IQ score > 3^41^), contrast analysis and point plots were used. *P* values less than 0.05 indicated statistical significance. The primary objective of this study was to provide evidence‐based support for clinical decision making by obstetricians. Therefore, we chose to use categorical variables as predictors in the main analysis, as this approach yields more intuitive results that are easier for obstetricians to understand and apply. For readers seeking more precise information, we have included results using continuous variables as predictors in the Supporting Information.

## 3 RESULTS

### 3.1 Participant characteristics

Overall, there were 30 035 term‐born participants with valid IQ scores at 5 years old (see Tables 1 and S1). A total of 3.84% (1.21% in MCS, 8.31% in LSAC, 5.62% in NLSY, and 4.47% in GUI, respectively) of covariates data were imputed (see Figure 1). Perinatal and sociodemographic characteristics by cohorts are summarized in Table 1. Of the 30 035 participants, 2111 (7.0%), 3796 (12.6%), 10 053 (33.5%), 8018 (26.7%), and 6057 (20.2%) were born at 37, 38, 39, 40, and 41 weeks, respectively. A total of 3054 (10.2%), 24 775 (82.5%), and 2206 (7.3%) were classified as SGA, AGA, and LGA, respectively, of which 50.7% of children were male, and the mean maternal age was 29.3, while 66.5% of children's mothers were married. A total of 88.8% of children spoke English as their first language, 24.5% of children's maternal education level was university and beyond, while 28.6% of children were living in a high‐income family. The number and percentage of children with an IQ *Z*‐score lower than −1 standard deviation (SD) across each week of gestation in term‐born children are shown in Table S3. A total of 731 (12.1%) of 6057 children born at 41 weeks had an IQ < –1 SD, whereas 330 (15.6%) of 2111 children born at 37 weeks had an IQ < –1 SD.

**TABLE 1:** Perinatal and demographic characteristics.

Characteristic	No. (%)				
	GUI (Ireland) (*n* = 7062)	LSAC (Australia) (*n* = 3507)	MCS (UK) (*n* = 12 939)	NLSY79 (USA) (*n* = 6527)	Total (*N* = 30 035)
Gestational age at term					
37 weeks	366 (5.2%)	237 (6.8%)	851 (6.6%)	657 (10.1%)	2111 (7.0%)
38 weeks	938 (13.3%)	704 (20.1%)	2003 (15.5%)	151 (2.3%)	3796 (12.6%)
39 weeks	1532 (21.7%)	760 (21.7%)	3050 (23.6%)	4711 (72.2%)	10 053 (33.5%)
40 weeks	2552 (36.1%)	1147 (32.7%)	4083 (31.6%)	236 (3.6%)	8018 (26.7%)
41 weeks	1674 (23.7%)	659 (18.8%)	2952 (22.8%)	772 (11.8%)	6057 (20.2%)
Gestational age at term, mean (SD), weeks	39.6 (1.14)	39.4 (1.19)	39.7 (1.20)	39.0 (0.966)	39.5 (1.16)
Birth weight at term, mean (SD), grams	3550 (454)	3490 (485)	3420 (499)	3390 (498)	3450 (491)
Fenton's birth weight percentiles					
<10th SGA	415 (5.9%)	274 (7.8%)	1601 (12.4%)	764 (11.7%)	3054 (10.2%)
10–90th AGA	6055 (85.7%)	2957 (84.3%)	10 584 (81.8%)	5179 (79.3%)	24 775 (82.5%)
>90th LGA	592 (8.4%)	276 (7.9%)	754 (5.8%)	584 (8.9%)	2206 (7.3%)
English as first language	6382 (90.4%)	3189 (90.9%)	11 800 (91.2%)	5299 (81.2%)	26 670 (88.8%)
Child male sex	3556 (50.4%)	1790 (51.0%)	6566 (50.7%)	3304 (50.6%)	15 216 (50.7%)
Maternal age, mean (SD), years	32.1 (5.15)	31.5 (5.06)	29.5 (5.85)	24.6 (5.55)	29.3 (6.15)
Maternal education					
Non‐university	3755 (53.2%)	1214 (34.6%)	10 663 (82.4%)	4621 (70.8%)	20 253 (67.4%)
University	1930 (27.3%)	1282 (36.6%)	2251 (17.4%)	1906 (29.2%)	7369 (24.5%)
Missing	1377 (19.5%)	1011 (28.8%)	25 (0.2%)	0 (0%)	2413 (8.0%)
Family household income					
Low	2224 (31.5%)	867 (24.7%)	4645 (35.9%)	1483 (22.7%)	9219 (30.7%)
Medium	2563 (36.3%)	1458 (41.6%)	4502 (34.8%)	3030 (46.4%)	11 553 (38.5%)
High	1784 (25.3%)	1015 (28.9%)	3763 (29.1%)	2014 (30.9%)	8576 (28.6%)
Missing	491 (7.0%)	167 (4.8%)	29 (0.2%)	0 (0%)	687 (2.3%)
Maternal height (cm), mean (SD)	164 (6.70)	165 (7.26)	164 (7.05)	162 (6.83)	164 (7.01)
Maternal weight (kg), mean (SD)	67.6 (12.5)	69.3 (15.2)	63.5 (12.4)	61.2 (13.0)	64.6 (13.2)
Mother married	5007 (70.9%)	2738 (78.1%)	8012 (61.9%)	4205 (64.4%)	19 962 (66.5%)
Full IQ, mean (SD), *Z*‐score	0 (1.00)	0 (1.00)	0 (1.00)	0 (1.00)	0 (1.00)

Abbreviations: AGA, appropriate for gestational age; GUI, Growing Up in Ireland; LGA, large for gestational age; LSAC, longitudinal study of Australian children; MCS, Millennium Cohort Study; NLSY79, National Longitudinal Survey of Youth 1979; SD, standard deviation; SGA, small for gestational age.

### 3.2 Main effects of gestation and fetal size category and their interactions

The overall adjusted model using categorical predictors is shown in Table 2, and the comparison between results with and without multiple imputation is presented in Table S4. There were statistically significant lower IQ *Z*‐scores in children born at 37 weeks (−0.09 [−0.15 to −0.04]), 38 weeks (−0.10 [−0.14 to −0.05]), 39 weeks (−0.05 [−0.08 to −0.01]), 40 weeks (−0.05 [−0.08 to −0.02]) compared with children born at 41 weeks. Overall, of the fetal size categories, SGA was negatively associated with IQ compared to AGA (IQ *Z*‐score: −0.13 [−0.20 to −0.06]). No statistically significant differences in IQ were found between LGA‐ and AGA‐born children. No statistically significant interactions were found between the fetal size category and gestational age at term. Sensitivity analysis using birth weight as continuous variable also indicated a main effect of both gestation and birth weight and no interaction between these two independent factors. The overall adjusted model using continuous predictors, as shown in Table S2, yielded consistent results with the one using categorical predictors. Table S2 further shows that higher family income, higher maternal education and English language spoken at home are strongly associated with higher IQ while female sex and mother being married having additional positive association with IQ.

**TABLE 2:** Results of linear regression analyses on IQ *Z*‐score in term children (categorical gestation age and fetal size category as predictors, *n* = 30 035).

Predictors	Gestational age and fetal size category only			With all covariates^a^		
	*β* estimate	95% CI	*P* value	*β* estimate	95% CI	*P* value
Gestational age						
37 weeks	**−0.10**	**−0.16 to −0.04**	**<0.001**	**−0.09**	**−0.15 to −0.04**	**<0.001**
38 weeks	**−0.12**	**−0.16 to −0.07**	**<0.001**	**−0.10**	**−0.14 to −0.05**	**<0.001**
39 weeks	**−0.06**	**−0.10 to −0.03**	**<0.001**	**−0.05**	**−0.08 to −0.01**	**0.005**
40 weeks	**−0.07**	**−0.11 to −0.03**	**<0.001**	**−0.05**	**−0.08 to −0.02**	**0.004**
41 weeks	Ref			Ref		
Fetal size category						
SGA	**−0.24**	**−0.31 to −0.16**	**<0.001**	**−0.13**	**−0.20 to −0.06**	**<0.001**
AGA	Ref			Ref		
LGA	0.02	−0.11 to 0.16	0.75	0.02	−0.10 to 0.15	0.71
37 weeks × SGA	−0.13	−0.30 to 0.04	0.13	−0.14	−0.29 to 0.02	0.09
38 weeks × SGA	−0.01	−0.15 to 0.13	0.86	−0.03	−0.16 to 0.10	0.66
39 weeks × SGA	−0.05	−0.15 to 0.05	0.35	−0.04	−0.14 to 0.05	0.36
40 weeks × SGA	−0.01	−0.12 to 0.09	0.82	0.02	−0.08 to 0.11	0.75
37 weeks × LGA	0.04	−0.14 to 0.23	0.64	0.04	−0.13 to 0.22	0.62
38 weeks × LGA	−0.01	−0.18 to 0.17	0.95	−0.06	−0.22 to 0.10	0.48
39 weeks × LGA	0.04	−0.12 to 0.19	0.64	−0.03	−0.17 to 0.12	0.73
40 weeks × LGA	0.06	−0.11 to 0.22	0.50	0.01	−0.15 to 0.17	0.89

Abbreviations: AGA, appropriate for gestational age; CI, confidence interval; IQ, intelligent quotient; LGA, large for gestational age; SGA, small for gestational age.

The *P* values represent significance for bold values.

^a^ Adjusted for child sex, maternal age, maternal height, maternal weight, marital status, child's primary language, maternal education, household income and cohort.

### 3.3 Conjoint associations between fetal size category, gestational age, and child IQ

**FIGURE 2:**
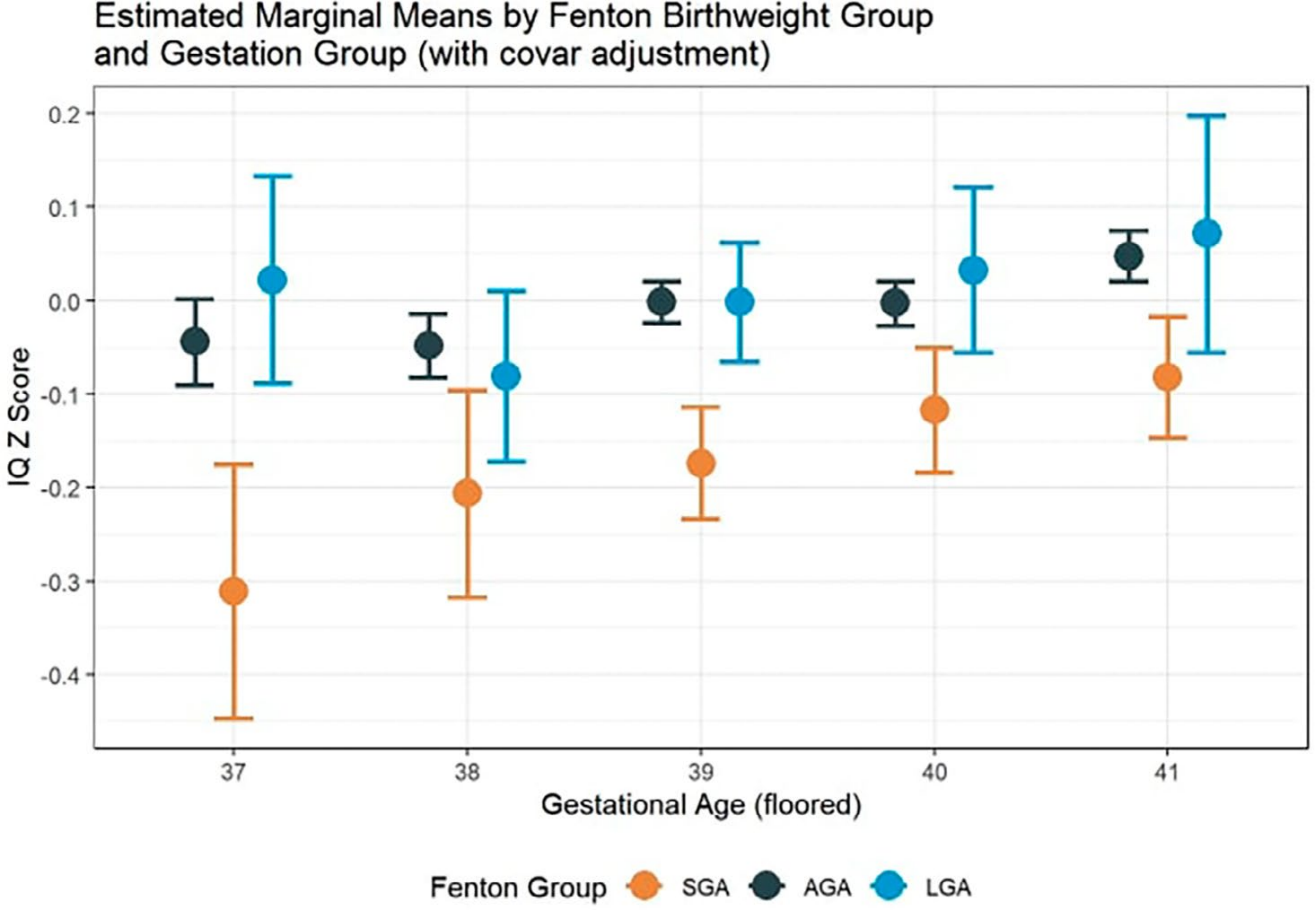
Adjusted IQ *Z*‐score at each gestation in term‐born children classified as LGA, AGA and SGA using Fenton reference. Adjusted for child sex, maternal age, maternal height, maternal weight, marital status, child's primary language, maternal education, household income and cohort. AGA, appropriate for gestational age; IQ, intelligent quotient; LGA, large for gestational age; SGA, small for gestational age.

Even though the results suggested no interaction between gestation and fetal size in term‐born children, conjoint associations between gestation, fetal size, and IQ were tested for clinical relevance. Figure 2 illustrates the conjoint associations between gestational age and IQ *Z*‐scores at age 5 in each fetal size category while controlling for the aforementioned confounders. There was a 0.06 IQ *Z*‐score decrease, but it was not statistically significant in LGA‐born children born at 37 weeks compared to those born at 41 weeks at age 5. However, compared with AGA‐born children born at 41 weeks (IQ *Z*‐score 0.05 [0.02 to 0.08]), AGA‐born children born at 37 weeks (−0.04 [−0.09 to 0.00]), 38 weeks (−0.05 [−0.08 to −0.02]), 39 weeks (0.00 [−0.02 to 0.02]), and 40 weeks (0.00 [−0.03 to 0.02]) had significantly lower IQ. Compared with SGA‐born children born at 41 weeks (−0.08 [−0.15 to −0.02]), SGA children at 37 weeks (−0.31 [−0.45 to −0.18]) had significantly lower IQ.

Figure 2 also illustrates the relationship between the fetal size category and IQ *Z*‐score at age 5, adjusted for confounders, in each gestational week at term. At each gestation from 37 to 41 weeks, compared with children born AGA, SGA was associated with lower IQ (IQ *Z*‐score in 37 weeks: −0.31 [−0.45 to −0.18] vs. −0.04 [−0.09 to 0.00]; 38 weeks: −0.21 [−0.32 to −0.10] vs. −0.05 [−0.08 to −0.02]; 39 weeks: −0.18 [−0.24 to −0.11] vs. 0.00 [−0.02 to 0.02]; 40 weeks: −0.12 [−0.18 to −0.05] vs. 0.00 [−0.03 to 0.02]; 41 weeks: −0.08 [−0.15 to −0.02] vs. 0.04 [0.02 to 0.08]). No statistically significant difference in IQ at age 5 was found between LGA‐born children and AGA‐born children born at any gestational age from 37 to 41 weeks.

### 3.4 IQ reduction by a clinically meaningful magnitude

Only a small clinically significant difference in IQ (i.e., 0.23 standard mean difference in IQ *Z*‐score × 15 points = 3.45 IQ difference) was found between SGA children born at 37 weeks and those born at 41 weeks (Table 3). No other reductions in IQ for the conjoint associations of gestational age and fetal size in term‐born children was clinically significant.

**TABLE 3:** Contrast analysis of gestational age at term within each fetal size category.

Fetal size category	Gestation comparison	Estimated IQ *Z*‐score difference (SE)	Adjusted *P* value^a^
SGA	37w vs. 41w	−0.23 (0.08)	0.01
SGA	38w vs. 41w	−0.13 (0.07)	0.10
SGA	39w vs. 41w	−0.11 (0.05)	0.04
SGA	40w vs. 41w	−0.08 (0.05)	0.15
AGA	37w vs. 41w	−0.10 (0.03)	0.001
AGA	38w vs. 41w	−0.12 (0.02)	<0.001
AGA	39w vs. 41w	−0.06 (0.02)	0.001
AGA	40w vs. 41w	−0.07 (0.02)	<0.001
LGA	37w vs. 41w	−0.06 (0.09)	0.64
LGA	38w vs. 41w	−0.12 (0.08)	0.19
LGA	39w vs. 41w	−0.03 (0.08)	0.80
LGA	40w vs. 41w	−0.01 (0.08)	0.88

Abbreviations: AGA, appropriate for gestational age; CI, confidence interval; IQ, intelligent quotient; LGA, large for gestational age; SE, standard error; SGA, small for gestational age.

^a^ Adjusted for child sex, maternal age, maternal height, maternal weight, marital status, child's primary language, maternal education, household income and cohort.

## 4 DISCUSSION

This study harmonized data from four national cohorts in Australia, UK, USA, and Ireland to investigate the association of gestational age and fetal size and their conjoint associations on intelligence in term‐born children. Gestational age before 41 weeks and SGA (but not LGA) had small adverse association on IQ at age 5 but there was no evidence of the associations of gestation at term on IQ been moderated by fetal size. Rather, SGA was associated with lower IQ at each gestational week in term‐born children compared to AGA. However, these effect sizes were small and did not reach clinical significance, as most of them were below 0.2 (3 IQ points).^41^ Only SGA children born at 37 weeks had a clinically meaningful IQ reduction at age 5 compared to those born at 41 weeks.

The finding that birth at 37 weeks compared to 41 weeks is associated with a 1.35‐point reduction in IQ (0.09 IQ *Z*‐scores) is essentially consistent with a previous meta‐analysis^14^ that suggested early‐term delivery (37 weeks) is associated with 1.95 lower IQ scores, a higher risk of cognitive impairment, and poorer academic performance. Although the 0.05 effect size between 40 and 41 weeks did not reach clinical significance, our findings are consistent with the results of the study by Mackey et al.^6^ that full‐term birth (39–40 weeks) before 41 weeks was associated with a slightly higher risk of special education needs, but contradict the conclusions that no difference was found in academic achievement in children born from 39 to 41 weeks by Wehby et al.^11^ Although IQ is highly heritable, a recent study of moderately preterm children^42^ reported that gestational age may exert a long‐term influence on cognitive development independent of genetic factors, highlighting the biological risks associated with shortened gestation.

The present study also indicates that compared with AGA, SGA is associated with 1.95 lower IQ scores (0.13 IQ *Z*‐scores), consistent in direction but smaller than the 5.10 lower IQ scores shown in a previous meta‐analysis.^15^ There are adverse associations of SGA on IQ regardless of the gestational age at birth among term‐born individuals, and early‐term delivery of SGA children can result in a clinically meaningful reduction in IQ. SGA‐born children include former constitutionally small fetuses and those with intrauterine growth restriction (IUGR), and the latter case^15^, ^43^ is associated with adverse cognitive outcomes. Given the current uncertainty about whether the intrauterine environment provides superior cognitive development conditions for SGA/IUGR children compared to early extrauterine exposure, our study suggests that early exposure to the extrauterine environment does not improve children's cognitive development outcomes.

Our results suggest that LGA does not have a significant adverse effect on cognitive development. A previous meta‐analysis^14^ suggested a slight beneficial effect, that is, 0.09 higher IQ scores (not replicated in our study). Together this suggests that, as a group, being large has not adverse effects on cognitive development due to good nutritional status. However, there may be other trade‐offs.^5^ While we know LGA is associated with increased risks of other conditions, such as metabolic and cardiovascular diseases, no evidence^14^ has been found linking LGA to a decreased cognitive score, an increased risk of cognitive impairment, or low academic performance. Additionally, LGA is associated with an increased risk (8.1%) of neonatal hypoglycemia.^44^ However, current evidence on the association between neonatal hypoglycemia and childhood cognitive development is conflicting: for example, a 2024 meta‐analysis^45^ suggested an adverse correlation between neonatal hypoglycemia and cognitive development, but the certainty of the evidence was very low. Conversely, a study^46^ published in JAMA found no association between neonatal hypoglycemia and academic performance in mid‐childhood.

The observation that shorter gestational age is negatively correlated with cognitive development, independent of fetal size, aligns with findings from previous studies.^18^, ^19^, ^21^ However, most studies did not specifically look at 37–41 weeks (the most common gestational age for selective induction or cesarean section). Furthermore, a study of preterm infants^23^ also found no interaction between preterm birth and SGA in influencing cognitive development. Conversely, some studies^20^, ^22^ have identified a significant interaction between absolute birth weight and gestational age. For instance, low birth weight is a risk factor for impaired IQ in term infants but not in preterm infants. However, this conclusion disregards the evident fact that absolute birth weight and gestational age exhibit a strong positive correlation. Thus, the conclusion that “fetal size and gestational age show no significant interaction with cognitive development” becomes more robust after adjusting for the effects of gestational age on birth weight (i.e., using birth weight for gestational age measurement instead).The results provide important information for mothers and obstetricians to make informed decisions when weighing small associations on cognitive development affecting many children versus reducing rare catastrophic consequences for babies of different sizes. For example, the results suggest that early induction of labor in LGA babies to reduce the risk of shoulder dystocia may have littleadverse long‐term associations on cognitive outcomes.

The present study has several strengths including large sample size and the resulting significant statistical power achieved by utilizing four national cohort studies. Observation studies currently provide the best available evidence, as there are no relevant large randomized controlled trials (RCTs) that have considered both short‐ and long‐term consequences.^4^ This study includes covariates that are strongly associated with IQ, such as maternal height^47^ and weight and maternal education,^48^ income, or single parenthood. Furthermore, this large, pooled sample used IQ assessed at 5 years of age as an outcome rather than assessments of cognitive abilities in infancy. Cognitive measures in infancy exhibit low stability, whereas IQ tests from 4 to 5 years demonstrate moderate to high stability over the next 5 years and into adulthood.^49^, ^50^ There are also limitations. First, birth weight and gestational age were reported by parents rather than captured from hospital records. However, based on studies by Tate et al.^34^ and Poulsen et al.,^35^ over 90% of parents can accurately recall their children's birth weight and gestational age within the first year after delivery. Second, the earlier births were a combination of spontaneous labor, cesarean sections, and inductions, including pregnancy complications such as gestational diabetes and hypertensive disorders. Due to a lack of data on indication for delivery, we could not rule out the confounding by indication for delivery. Third, as no data on neonatal complications or prenatal exposures could be harmonized across all four cohorts, we were unable to exclude residual confounding regarding these factors. Fourth, in the absence of antenatal fetal size measurements, we used birth weight for gestation as a substitute. However, birth weight is an accurate measurement and approximately equal to the weight of the fetus in the mother's uterus at the time of delivery.

We recommend two directions for future research. First, RCTs are needed to address causal questions unresolved by existing observational studies: specifically, how planned early induction of labor compared to standard care affects long‐term cognitive outcomes. Second, explore methods to enhance the accuracy of clinical diagnosis. Although birth weight used in our study is the most accurate measure of fetal size, clinical practice relies solely on prenatal estimates.^24^, ^43^, ^51^ Previous research^24^ has shown significant limitations in the accuracy of prenatal ultrasound as a measure of fetal size. Therefore, there is an urgent need for low‐cost, highly accurate alternative methods to assist obstetricians in clinical decision making.

In conclusion, this investigation, utilizing four national cohorts totaling more than 30 000 term‐born children, found that the association between gestation and child IQ at age 5 was not moderated by fetal size. Regardless of gestational age at birth, SGA (but not LGA) is consistently and unfavorably associated with poorer cognitive outcomes in term‐born children. SGA children's birth at 37 weeks were associated with a clinically reelvant reduction in IQ at age 5. RCTs are needed to determine the effect of clinical interventions on delivery timing in newborns across different fetal size categories on long‐term cognitive outcomes.

## AUTHOR CONTRIBUTIONS

XZ, SQ and DW designed the study and wrote the analysis plan. RE and SL obtained the data from four national cohorts. RE conducted the analysis. XZ, RE, SL, SQ, and DW reviewed and interpreted the results. XZ drafted the article. DW, RE, SQ, and SL reviewed the draft article and provided feedback. All authors read and approved the final version for publication.

## FUNDING INFORMATION

Xuan Zhao is supported by the Chancellor's International Scholarship of the University of Warwick and the BB2UP PhD Fellowship (supported through a legacy gift from University of Warwick alumnus Jack Straw (BSc Mathematics and Economics, 1969‐72)); Siobhan Quenby is supported by the BB2UP grant; Dieter Wolke is also supported by the BB2UP grant and additionally by a UKRI frontier research grant (EP/X023206/1) under the UK government's funding guarantee for ERC‐AdG grants; Sakari Lemola was supported by the NORFACE Joint Research Program on Dynamics of Inequality Across the Life‐course, which was cofunded by the European Commission through Horizon 2020 under grant agreement no. 724363 (grant no. 462‐16‐040).

## CONFLICT OF INTEREST STATEMENT

All authors confirm no conflicts of interest. All funders had no role in the design and conduct of the study.

## Supporting information


**Table S1.** Systematic drop‐out test in four cohorts.

**Table S2.** Results of linear regression analysis on IQ *Z*‐score in term‐born children (continuous gestation age and fetal size as predictors, *n* = 30 035).

**Table S3.** Number and percentage of children had intelligence quotient *Z*‐score lower than −1 standard deviation across each week of gestation in term‐born children.

**Table S4.** Comparison of results with and without imputation.

## Data Availability

The data used in this publication is open access but must be requested from each cohort's respective websites: MCS: https://cls.ucl.ac.uk/cls‐studies/millennium‐cohort‐study/. GUI: https://www.growingup.gov.ie/. LSAC: https://growingupinaustralia.gov.au/. NLSY79: https://www.nlsinfo.org/content/cohorts/nlsy79‐children.

## References

[1] Chauhan SP , Ananth CV . Induction of labor in the United States: a critical appraisal of appropriateness and reducibility. Semin Perinatol. 2012;36(5):336‐343. 23009965 10.1053/j.semperi.2012.04.016

[2] Murthy K , Grobman WA , Lee TA , Holl JL . Trends in induction of labor at early‐term gestation. Am J Obstet Gynecol. 2011;204(5):435.e1‐435.e6. 10.1016/j.ajog.2010.12.023 21345413

[3] Chrissie Y . UK C‐Section Rates 2023: Stats, Perspectives & Guidance 2023. 2023. Available from: https://www.chrissieyu.com/c‐section‐rates‐statistics‐uk‐global/

[4] Middleton P , Shepherd E , Morris J , Crowther CA , Gomersall JC . Induction of labour at or beyond 37 weeks' gestation. Cochrane Database Syst Rev. 2020;7(7):CD004945. 32666584 10.1002/14651858.CD004945.pub5 PMC7389871

[5] Wolke D , Zhao X , Quenby S . The trade‐off of delivery timing between reduced perinatal complications versus adverse long‐term outcomes. Paediatr Perinat Epidemiol. 2025;39(4):370‐372. 40254800 10.1111/ppe.70025 PMC12121327

[6] MacKay DF , Smith GCS , Dobbie R , Pell JP . Gestational age at delivery and special educational need: retrospective cohort study of 407,503 schoolchildren. PLoS Med. 2010;7(6):e1000289. 20543995 10.1371/journal.pmed.1000289 PMC2882432

[7] Rose O , Blanco E , Martinez SM , et al. Developmental scores at 1 year with increasing gestational age, 37‐41 weeks. Pediatrics. 2013;131(5):e1475‐e1481. 23589812 10.1542/peds.2012-3215 PMC3639464

[8] Espel EV , Glynn LM , Sandman CA , Davis EP . Longer gestation among children born full term influences cognitive and motor development. PLoS One. 2014;9(11):e113758. 25423150 10.1371/journal.pone.0113758 PMC4244187

[9] Hua J , Sun J , Cao Z , et al. Differentiating the cognitive development of early‐term births in infants and toddlers: a cross‐sectional study in China. BMJ Open. 2019;9(4):e025275. 10.1136/bmjopen-2018-025275 PMC6500364 30975675

[10] Richards JL , Drews‐Botsch C , Sales JM , Flanders WD , Kramer MR . Describing the shape of the relationship between gestational age at birth and cognitive development in a nationally representative U.S. birth cohort. Paediatr Perinat Epidemiol. 2016;30(6):571‐582. 27781289 10.1111/ppe.12319 PMC5134736

[11] Wehby GL . Association between gestational age and academic achievement of children born at term. JAMA Netw Open. 2023;6(7):e2326451. 37523180 10.1001/jamanetworkopen.2023.26451 PMC10391305

[12] Adanikin A , Lawlor DA , Pell JP , Nelson SM , Smith GCS , Iliodromiti S . Association of birthweight centiles and early childhood development of singleton infants born from 37 weeks of gestation in Scotland: a population‐based cohort study. PLoS Med. 2022;19(10):e1004108. 36219591 10.1371/journal.pmed.1004108 PMC9553050

[13] Khambalia AZ , Algert CS , Bowen JR , Collie RJ , Roberts CL . Long‐term outcomes for large for gestational age infants born at term. J Paediatr Child Health. 2017;53(9):876‐881. 28868781 10.1111/jpc.13593

[14] Zhao X , Poskett A , Stracke M , Quenby S , Wolke D . Cognitive and academic outcomes of large‐for‐gestational‐age babies born at early term: a systematic review and meta‐analysis. Acta Obstet Gynecol Scand. 2024;104:288‐301. 39475202 10.1111/aogs.15001 PMC11782071

[15] Sacchi C , Marino C , Nosarti C , Vieno A , Visentin S , Simonelli A . Association of intrauterine growth restriction and small for gestational age status with childhood cognitive outcomes: a systematic review and meta‐analysis. JAMA Pediatr. 2020;174(8):772‐781. 32453414 10.1001/jamapediatrics.2020.1097 PMC7251506

[16] Gale‐Grant O , Fenn‐Moltu S , França LGS , et al. Effects of gestational age at birth on perinatal structural brain development in healthy term‐born babies. Hum Brain Mapp. 2022;43(5):1577‐1589. 34897872 10.1002/hbm.25743 PMC8886657

[17] Cabal‐Herrera A , Kigen B , Kapanga E , Samia A , Nabwera H , Samia P . The impact of undernutrition and overnutrition on early brain development. Semin Pediatr Neurol. 2025;55:101212. 41076301 10.1016/j.spen.2025.101212

[18] Eves R , Mendonça M , Bartmann P , Wolke D . Small for gestational age‐cognitive performance from infancy to adulthood: an observational study. BJOG. 2020;127(13):1598‐1606. 32479707 10.1111/1471-0528.16341

[19] Eves R , Wolke D , Spiegler J , Lemola S . Association of birth weight centiles and gestational age with cognitive performance at age 5 years. JAMA Netw Open. 2023;6(8):e2331815. 37651137 10.1001/jamanetworkopen.2023.31815 PMC10472194

[20] Eide MG , Oyen N , Skjaerven R , Bjerkedal T . Associations of birth size, gestational age, and adult size with intellectual performance: evidence from a cohort of Norwegian men. Pediatr Res. 2007;62(5):636‐642. 17805203 10.1203/PDR.0b013e31815586e9

[21] Yang S , Platt RW , Kramer MS . Variation in child cognitive ability by week of gestation among healthy term births. Am J Epidemiol. 2010;171(4):399‐406. 20080810 10.1093/aje/kwp413 PMC3435092

[22] Lagerström M , Bremme K , Eneroth P , Magnusson D . School performance and IQ‐test scores at age 13 as related to birth weight and gestational age. Scand J Psychol. 1991;32(4):316‐324. 1775949 10.1111/j.1467-9450.1991.tb00882.x

[23] Kong L , Nivins S , Chen X , Liang Y , Gissler M , Lavebratt C . Association of preterm birth and birth size status with neurodevelopmental and psychiatric disorders in spontaneous births. Eur Child Adolesc Psychiatry. 2025;34(1):261‐273. 38866929 10.1007/s00787-024-02489-5 PMC11805797

[24] Gardosi J , Ewington LJ , Booth K , et al. Induction of labour versus standard care to prevent shoulder dystocia in fetuses suspected to be large for gestational age in the UK (the Big Baby trial): a multicentre, open‐label, randomised controlled trial. Lancet. 2025;405(10491):1743‐1756. 40319899 10.1016/S0140-6736(25)00162-X

[25] Connelly R , Platt L . Cohort profile: UK Millennium Cohort Study (MCS). Int J Epidemiol. 2014;43(6):1719‐1725. 24550246 10.1093/ije/dyu001

[26] Soloff Carol LD , Johnstone R . The longitudinal study of Australian children: an Australian Government initiative sample design contents. 2005.

[27] Rothstein DS , Carr D , Cooksey E . Cohort profile: the National Longitudinal Survey of youth 1979 (NLSY79). Int J Epidemiol. 2019;48(1):22‐e. 29982488 10.1093/ije/dyy133 PMC6380301

[28] James Williams SG , McNally S , Murray A , Quail A . Growing up in Ireland national longitudinal study of children. The infants and their families. 2012.

[29] Meeus W , Vollebergh W , Branje S , et al. On imbalance of impulse control and sensation seeking and adolescent risk: an intra‐individual developmental test of the dual systems and maturational imbalance models. J Youth Adolesc. 2021;50(5):827‐840. 33745073 10.1007/s10964-021-01419-x PMC8043917

[30] Kelly Y , Sacker A , Del Bono E , Francesconi M , Marmot M . What role for the home learning environment and parenting in reducing the socioeconomic gradient in child development? Findings from the Millennium Cohort Study. Arch Dis Child. 2011;96(9):832‐837. 21666278 10.1136/adc.2010.195917

[31] Shiely F , Ng HY , Berkery EM , Murrin C , Kelleher C , Hayes K . The association between weight perception and BMI: report and measurement data from the growing up in Ireland cohort study of 9‐year olds. Int J Obes. 2017;41(1):46‐53. 10.1038/ijo.2016.162 27671034

[32] Christensen D , Zubrick SR , Lawrence D , Mitrou F , Taylor CL . Risk factors for low receptive vocabulary abilities in the preschool and early school years in the longitudinal study of Australian children. PLoS One. 2014;9(7):e101476. 24988308 10.1371/journal.pone.0101476 PMC4079336

[33] Fenton TR , Kim JH . A systematic review and meta‐analysis to revise the Fenton growth chart for preterm infants. BMC Pediatr. 2013;13:59. 23601190 10.1186/1471-2431-13-59 PMC3637477

[34] Tate AR , Dezateux C , Cole TJ , Davidson L , Millennium Cohort Study Child Health G . Factors affecting a mother's recall of her baby's birth weight. Int J Epidemiol. 2005;34(3):688‐695. 15737964 10.1093/ije/dyi029

[35] Poulsen G , Kurinczuk JJ , Wolke D , et al. Accurate reporting of expected delivery date by mothers 9 months after birth. J Clin Epidemiol. 2011;64(12):1444‐1450. 21684117 10.1016/j.jclinepi.2011.03.007

[36] Elliott CD , Smith P , McCulloch K . BAS II: British Ability Scales Second Edition. NFER‐Nelson; 1996.

[37] O'Donnell L . The Wechsler intelligence scale for children—fourth edition. In: Naglieri JA , Goldstein S , eds. Practitioner's Guide to Assessing Intelligence and Achievement. John Wiley & Sons Inc; 2009.

[38] Dunn LM , Markwardt FC . Peabody Individual Achievement Test. American Guidance Service, Incorporated; 1970.

[39] Dunn LM . Examiner's Manual for the PPVT‐III, Peabody Picture Vocabulary Test, Third Edition. American Guidance Service; 1997.

[40] Vance HR(B) . Peabody Picture Vocabulary Test‐Revised (PPVT–R). Diagnostique. 1989;15(1–4):149‐160.

[41] Cohen J . Statistical Power Analysis for the Behavioral Sciences. 2nd ed. Lawrence Erlbaum Associates; 1988.

[42] Nivins S , Padilla N , Kvanta H , Ådén U . Gestational age and cognitive development in childhood. JAMA Netw Open. 2025;8(4):e254580. 40227687 10.1001/jamanetworkopen.2025.4580 PMC11997729

[43] Rogne T , Engstrøm AA , Jacobsen GW , Skranes J , Østgård HF , Martinussen M . Fetal growth, cognitive function, and brain volumes in childhood and adolescence. Obstet Gynecol. 2015;125(3):673‐682. 25730232 10.1097/AOG.0000000000000694

[44] Holtrop PC . The frequency of hypoglycemia in full‐term large and small for gestational age newborns. Am J Perinatol. 1993;10(2):150‐154. 8476480 10.1055/s-2007-994649

[45] Diggikar S , Trif P , Mudura D , et al. Neonatal hypoglycemia and neurodevelopmental outcomes—an updated systematic review and meta‐analysis. Life. 2024;14(12):1618. 39768326 10.3390/life14121618 PMC11677687

[46] Shah R , Dai DWT , Alsweiler JM , et al. Association of neonatal hypoglycemia with academic performance in mid‐childhood. JAMA. 2022;327(12):1158‐1170. 35315886 10.1001/jama.2022.0992 PMC8941348

[47] Gustafsson A , Bonnevier A , Kallen K . Association between small‐for‐gestational age and poor school performance in 2 500 000 children born 1973–2002. Acta Paediatr. 2024; 113(2):221‐228. 37950526 10.1111/apa.17037

[48] Bilsteen JF , Alenius S , Bråthen M , et al. Gestational age, parent education, and education in adulthood. Pediatrics. 2022;149(1):e2021051959. 34877601 10.1542/peds.2021-051959 PMC9645686

[49] Breeman LD , Jaekel J , Baumann N , Bartmann P , Wolke D . Preterm cognitive function into adulthood. Pediatrics. 2015;136(3):415‐423. 26260714 10.1542/peds.2015-0608

[50] Breit M , Scherrer V , Tucker‐Drob EM , Preckel F . The stability of cognitive abilities: a meta‐analytic review of longitudinal studies. Psychol Bull. 2024;150(4):399‐439. 38330347 10.1037/bul0000425 PMC11626988

[51] Ong YY , Ng NBH , Michael N , et al. Associations of fetal and postnatal growth trajectories with child cognition: the GUSTO cohort study. Int J Epidemiol. 2024;54(1):dyaf012. 39947656 10.1093/ije/dyaf012 PMC11825177

