# Supplementary material for: Fetal size, gestational age, and cognitive performance at 5 years in term‐born children: Four national cohorts' study
Source: Int J Gynaecol Obstet. 2025 Nov 17;173(2):791–800. doi: 10.1002/ijgo.70671 (PMC13094675; doi:10.1002/ijgo.70671)
Supplement: Supplementary file 1 — Table S1. Systematic drop‐out test in four cohorts. [file IJGO-173-791-s004.docx]

**Table S1**

Systematic drop-out test in 4 cohorts

|  | MCS | | | LSAC | | | NLSY79 | | | GUI | | |
| --- | --- | --- | --- | --- | --- | --- | --- | --- | --- | --- | --- | --- |
|  | OR | 95%CI | P value | OR | 95%CI | P value | OR | 95%CI | P value | OR | 95%CI | P value |
| Birthweight | 0.95 | 0.91 – 0.98 | **0.005** | 0.84 | 0.78 – 0.91 | **<0.001** | 0.98 | 0.92 – 1.03 | 0.413 | 0.87 | 0.82 – 0.93 | **<0.001** |
| Gestational weeks | 0.95 | 0.92 – 0.98 | **0.001** | 0.94 | 0.88 – 0.99 | **0.031** | 1.03 | 0.97 – 1.09 | 0.327 | 0.96 | 0.92 – 1.01 | 0.128 |
| Observations | 16351 | | | 4463 | | | 7962 | | | 8792 | | |
| R^2^ Tjur | 0.001 | | | 0.005 | | | 0.000 | | | 0.002 | | |

MCS: Millennium Cohort Study; LSAC: Longitudinal Study of Australian Children; NLSY79: National Longitudinal Survey of Youth 1979; GUI: Growing up in Ireland Study; OR: odds ratio; CI: confidence interval
