# Supplementary material for: Fetal size, gestational age, and cognitive performance at 5 years in term‐born children: Four national cohorts' study
Source: Int J Gynaecol Obstet. 2025 Nov 17;173(2):791–800. doi: 10.1002/ijgo.70671 (PMC13094675; doi:10.1002/ijgo.70671)
Supplement: Supplementary file 2 — Table S2. Results of linear regression analysis on IQ Z‐score in term‐born children (continuous gestation age and fetal size as predictors, n = 30 035). [file IJGO-173-791-s002.docx]

**Table S2**

Results of linear regression analysis on IQ z score in term children (continuous gestation age and birthweight z score as predictors, n = 30035)

|  | Gestational weeks and Birthweight Z score (Fenton) only | | | With all covariates | | |
| --- | --- | --- | --- | --- | --- | --- |
| Predictors | β estimate | 95% CI | P value | β estimate | 95% CI | P value |
| Gestational weeks | 0.04 | 0.03-0.05 | <0.001 | 0.03 | 0.02 – 0.04 | <0.001 |
| Birthweight Z score | 0.10 | 0.09-0.12 | <0.001 | 0.06 | 0.04 – 0.07 | <0.001 |
| Gestational weeks*Birthweight Z score interaction | -0.00 | -0.01-0.01 | 0.702 | -0.00 | -0.01 – 0.01 | 0.375 |
| Maternal height (cm) |  |  |  | 0.00 | 0.00 – 0.01 | <0.001 |
| Maternal weight (kg) |  |  |  | -0.00 | -0.00 – -0.00 | <0.001 |
| Medium income (ref = low) |  |  |  | 0.24 | 0.21 – 0.26 | <0.001 |
| High income (ref = low) |  |  |  | 0.41 | 0.38 – 0.44 | <0.001 |
| Maternal age (years) |  |  |  | 0.00 | 0.00 – 0.01 | <0.001 |
| Maternal education-University (ref = non-university) |  |  |  | 0.26 | 0.23 – 0.29 | <0.001 |
| Maternal first language is not English (ref = English) |  |  |  | -0.54 | -0.57 – -0.50 | <0.001 |
| Maternal non-married (ref = married) |  |  |  | -0.10 | -0.13 – -0.07 | <0.001 |
| Child’s sex Male (ref = female) |  |  |  | -0.13 | -0.15 – -0.10 | <0.001 |
| Cohort LSAC (ref = GUI) |  |  |  | -0.05 | -0.09 – -0.01 | 0.012 |
| Cohort MCS (ref = GUI) |  |  |  | 0.05 | 0.03 – 0.08 | <0.001 |
| Cohort NLSY79 (ref = GUI) |  |  |  | 0.07 | 0.03 – 0.10 | <0.001 |

Abbreviations: GUI, Growing Up in Ireland; LSAC, Longitudinal Study of Australian Children; MCS, Millennium Cohort Study; NLSY79, National Longitudinal Survey of Youth 1979; CI: confidence interval; IQ: intelligence quotient
