# Supplementary material for: Fetal size, gestational age, and cognitive performance at 5 years in term‐born children: Four national cohorts' study
Source: Int J Gynaecol Obstet. 2025 Nov 17;173(2):791–800. doi: 10.1002/ijgo.70671 (PMC13094675; doi:10.1002/ijgo.70671)
Supplement: Supplementary file 3 — Table S3. Number and percentage of children had intelligence quotient Z‐score lower than −1 standard deviation across each week of gestation in term‐born children. [file IJGO-173-791-s001.docx]

**Table S3**

Number and percentage of children with intelligence quotient Z score lower than -1 standard deviation across each week of gestation at term

| Gestational age (weeks) | IQ Z score <-1 SD (N, %) | | Total |
| --- | --- | --- | --- |
|  | No | Yes |  |
| 37 | 1781 (84.4) | 330 (15.6) | 2111 |
| 38 | 3237 (85.3) | 559 (14.7) | 3796 |
| 39 | 8552 (85.1) | 1501 (14.9) | 10053 |
| 40 | 6903 (86.1) | 1115 (13.9) | 8018 |
| 41 | 5326 (87.9) | 731 (12.1) | 6057 |
| Total | 25799 (85.9) | 4236 (14.1) | 30035 |

IQ, intelligence quotient; SD, standard deviation
