# Supplementary material for: Fetal size, gestational age, and cognitive performance at 5 years in term‐born children: Four national cohorts' study
Source: Int J Gynaecol Obstet. 2025 Nov 17;173(2):791–800. doi: 10.1002/ijgo.70671 (PMC13094675; doi:10.1002/ijgo.70671)
Supplement: Supplementary file 4 — Table S4. Comparison of results with and without imputation. [file IJGO-173-791-s003.docx]

**Table S4**

**Comparison of results with and without imputation**

|  | Without imputation ^a^ | | | With imputation ^a^ | | |
| --- | --- | --- | --- | --- | --- | --- |
| Predictors | β estimate | 95% CI | P value | β estimate | 95% CI | P value |
| Gestational age |  |  |  |  |  |  |
| 37 weeks | **-0.07** | **-0.13 – -0.01** | **0.029** | **-0.09** | **-0.15 – -0.04** | **<0.001** |
| 38 weeks | **-0.09** | **-0.13 – -0.04** | **<0.001** | **-0.10** | **-0.14 – -0.05** | **<0.001** |
| 39 weeks | **-0.04** | **-0.08 – -0.01** | **0.027** | **-0.05** | **-0.08 – -0.01** | **0.005** |
| 40 weeks | **-0.04** | **-0.08 – -0.00** | **0.028** | **-0.05** | **-0.08 – -0.02** | **0.004** |
| 41 weeks | Ref |  |  | Ref |  |  |
| Fetal size category |  |  |  |  |  |  |
| SGA | **-0.13** | **-0.20 – -0.05** | **<0.001** | **-0.13** | **-0.20 – -0.06** | **<0.001** |
| AGA | Ref |  |  | Ref |  |  |
| LGA | -0.01 | -0.16 – 0.13 | 0.85 | 0.02 | -0.10 – 0.15 | 0.71 |
| 37 weeks*SGA | **-0.21** | **-0.38 – -0.03** | **0.02** | -0.14 | -0.29 – 0.02 | 0.09 |
| 38 weeks*SGA | -0.06 | -0.21 – 0.08 | 0.40 | -0.03 | -0.16 – 0.10 | 0.66 |
| 39 weeks*SGA | -0.03 | -0.14 – 0.07 | 0.56 | -0.04 | -0.14 – 0.05 | 0.36 |
| 40 weeks*SGA | 0.02 | -0.09 – 0.13 | 0.73 | 0.02 | -0.08 – 0.11 | 0.75 |
| 37 weeks*LGA | 0.04 | -0.17 – 0.24 | 0.72 | 0.04 | -0.13 – 0.22 | 0.62 |
| 38 weeks*LGA | -0.01 | -0.19 – 0.16 | 0.88 | -0.06 | -0.22 – 0.10 | 0.48 |
| 39 weeks*LGA | 0.02 | -0.15 – 0.18 | 0.84 | -0.03 | -0.17 – 0.12 | 0.73 |
| 40 weeks*LGA | 0.04 | -0.13 – 0.22 | 0.65 | 0.01 | -0.15 – 0.17 | 0.89 |

Abbreviations: IQ: intelligent quotient; CI: confidence interval; SGA: small for gestational age; AGA: appropriate for gestational age; LGA: large for gestational age.

^a^ Adjusted for child sex, maternal age, maternal height, maternal weight, marital status, child’s primary language, maternal education, household income and cohort
